# Perceived Environmental Dynamism Promotes Entrepreneurial Team Member’s Innovation: Explanations Based on the Uncertainty Reduction Theory

**DOI:** 10.3390/ijerph18042033

**Published:** 2021-02-19

**Authors:** Xiao Deng, Xi Guo, Yenchun Jim Wu, Min Chen

**Affiliations:** 1 Business School, China University of Political Science and Law, Beijing 100088, China; xiaod@cupl.edu.cn (X.D.); gx0620@cupl.edu.cn (X.G.); 2 Graduate Institute of Global Business and Strategy, National Taiwan Normal University, Taipei 10645, Taiwan; ycwu@ntnu.edu.tw; 3 Academy of Financial Research, School of Business, Wenzhou University, Wenzhou 325035, China

**Keywords:** entrepreneurial team, environmental dynamism, individual innovation, uncertainty reduction theory, information exchange behavior

## Abstract

This study aims to examine the effect of perceived environmental dynamism on entrepreneurial team member’s innovation. Based on the uncertainty reduction theory, this study constructs a multilevel moderated mediation model of the relationship between perceived environmental dynamism and entrepreneurial team member’s innovation. By collecting questionnaires from 117 entrepreneurial team leaders and 479 team members in China, this research found that perceived environmental dynamism could stimulate entrepreneurial team members’ innovation via triggering their information exchange behavior. In addition, entrepreneurial team members’ intolerance for uncertainty and team cooperative climate can moderate the indirect positive relationship between perceived environmental dynamism and individual innovation. Our findings contribute to a better understanding of entrepreneurial team members’ responses to dynamic environment and their innovation behavior.

## 1. Introduction

Compared to teams in mature organizations, high external environmental dynamism is one of the most prominent traits of entrepreneurial teams [1,2,3]. Since entrepreneurial teams have an exceptionally flat organizational structure [4], members usually assume multiple roles and have more opportunities to be in direct contact with customers and markets on the frontline [5]; thus, they have a clearer perception of environmental dynamism. However, the effect of highly perceived environmental dynamism on their behavior remains unknown. Since individual innovative behavior among members is critical to the survival and development of entrepreneurial teams [6,7,8], this study attempts to investigate the relationship between external environmental dynamism, as perceived by entrepreneurial team members, and their innovative behavior. The aim is to better understand environmental dynamism’s effect on entrepreneurial team members, and to help them cope with external dynamic environments and improve individual innovation.

To investigate these effects, this study introduces the uncertainty reduction theory, which holds that since uncertainty evokes discomfort and anxiety, individuals are strongly motivated to engage in specific behaviors to reduce it [9,10]. Dynamism is a key characteristic of an environment that indicates a degree of rapid, unpredictable, and turbulent change [11]. In highly dynamic work situations, “there is rapid and discontinuous change in demand, competitors, technology and/or regulation, such that information is often inaccurate, unavailable, or obsolete” [12]. When members in an entrepreneurial team perceive that they are in a fully dynamic external environment, they may feel that they lack the accurate information to make correct decisions, which leads to a decline in the team’s predictive power, increasing feelings of uncertainty [13]. According to the uncertainty reduction theory [14], entrepreneurial team members that perceive high environmental dynamism have a stronger motivation to reduce uncertainty via action. 

During field interviews with entrepreneurial team members, information exchange behaviors—the act of exchanging work-related information, knowledge, and ideas, such as “exchanging ideas with colleagues” and “exchanging ideas with team leaders”—was repeatedly mentioned as the preferred strategy for reducing uncertainty [15,16]. Since some studies propose that information exchange effectively reduces individual uncertainty [17], this study suggests entrepreneurial team members, who perceive high environmental dynamism, should frequently engage in this behavior. In doing so, individuals can obtain more information [18,19] and expand their ideas to develop [20], improve, and implement ideas. Thus, individual innovation can be improved [21]. 

The uncertainty reduction theory also suggests that individuals have varying perceptions of uncertainty and their choices regarding uncertainty-reducing behavior in different situations [9]. Intolerance for uncertainty is an important individual variable that influences individuals’ processing of and responses to information about highly uncertain environments [22]. In a dynamic working environment, individuals with a high intolerance for uncertainty are often prone to negative emotions, like anxiety, and are unable to engage in appropriate behaviors that allow them to cope with the environment. Therefore, this study opines that intolerance for uncertainty among entrepreneurial team members affects their coping behaviors and subsequent behavioral outcomes.

Team cooperative climate emphasizes that a mutual sense of help and cooperation is incredibly important for a team’s success [23]. In a team with a high cooperative climate, individuals that engage in information exchange behavior will receive better feedback, more recognition, and bear lower social costs. In an entrepreneurial team with a high cooperative climate, this study poses that members who perceive a high environmental dynamism will adopt information exchange behavior more frequently to reduce uncertainty, improving subsequent individual innovation. Contrariwise, in a team with a low cooperative climate, information exchange behavior is often discouraged, causing members that perceive a high environmental dynamism to adopt information exchange behaviors less frequently to reduce uncertainty, which also affects subsequent innovative behavior. 

**Figure 1:**
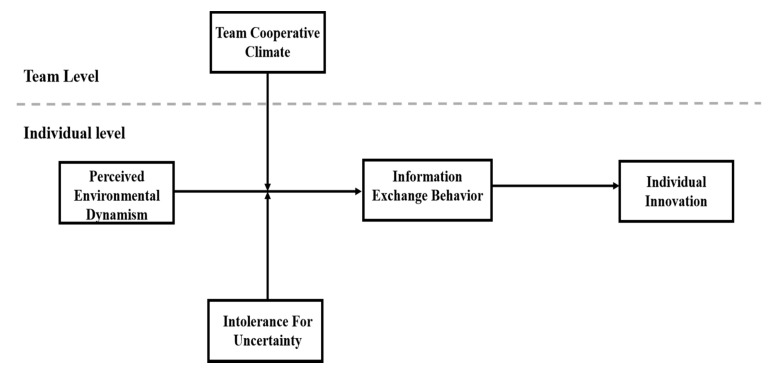
This study’s theoretical framework.

Figure 1 illustrates the theoretical framework of this study. In the following, we will first develop the hypothesis in the next section. Additionally, we will introduce our research design and the results of our data. Then we will discuss the contributions and future directions of this study.

## 2. Theories and Hypotheses

### 2.1. Information Exchange Behavior: A Mediating Effect

Based on the uncertainty reduction perspective, this study opines that individual’s information exchange behavior increases with environmental dynamism. First, entrepreneurial team members that have a high perception of environmental dynamism believe that as the market information changes rapidly, they lack the required information for decision-making and accurate behavior [24], increasing their feeling of uncertainty. By taking information exchange behavior, team members can acquire more work-related knowledge and others’ suggestions [20,25], which can provide more information for individuals to reduce uncertainty. Second, due to the increasing difficulty of decision-making under dynamic environment, entrepreneurial team members usually lack self-confidence in their behavior and decision-making skills [26], which further increases their feeling of uncertainty. Exchanging information in teams can promote interpersonal cooperation and support [27], improving individuals’ confidence and an affirmation of their judgment and ideas [27]. Thus, entrepreneurial team members tend to exchange more information with other team members when they perceive high environmental dynamism. This study proposes the following:

**Hypothesis** **1.** *Entrepreneurial team members’ perceived environmental dynamism is positively related to their information exchange behavior.*

The individual innovation process among team members is comprised of three stages: Idea generation, screening, and modification [28]. This study opines that individual’s information exchange behavior plays a role in promoting the three stages of individual innovation. First, entrepreneurial team members can obtain more varied information and ideas through information exchange, which provides them with additional raw materials for generating new and innovative ideas [20,29,30]. Second, sharing personal ideas also helps them improve understanding about others’ comments and suggestions regarding their ideas [20], enabling them to better screen and improve their innovative ideas. Lastly, information exchange behavior can promote trust and cooperation among team members [20,31], which may allow for the realization of innovative ideas [32]. Thus, this study proposes the following:

**Hypothesis** **2.** *Entrepreneurial team members’ information exchange behavior is positively related to their innovation.*

When entrepreneurial team members perceive that the work environment is dynamic, they will feel higher uncertainty about their decisions and performance. According to the uncertainty reduction theory, they tend to ask others to collect and confirm their information before making decision to reduce the uncertainty. With more exchanged information, they can produce innovative outcomes. Thus, this study proposes that member’s information exchange behavior can mediate the influence of environmental dynamism on member’s innovation.

**Hypothesis** **3.** *Entrepreneurial team members’ information exchange behavior can mediate the positive relationship between perceived environmental dynamism and their innovation.*

### 2.2. Intolerance for Uncertainty: A Moderating Effect

An individual’s trait of intolerance for uncertainty refers to the degree that an individual responds to negative emotions, perceptions, and behaviors when encountering uncertain situations [22]. Studies find that people with a high intolerance for uncertainty are not only more likely to notice uncertain factors in a particular situation and even amplify their effects, but also demonstrate more negative reactions, such as “I cannot sleep soundly when I feel uncertain” and “I cannot do other things when I feel uncertain” [33]. Berenbaum et al. [34] find that people with a high intolerance for uncertainty are easily overwhelmed by negative feelings in a highly uncertain environment; thus, they can only experience negative emotions—like feeling miserable and anxious—but are unable to respond to other clues in the environment and engage in uncertainty-reducing behavior. As a result, entrepreneurial team members with a high intolerance for uncertainty often pay too much attention to an environment’s uncertainty, overestimating its negative aspects and experiencing negative emotions, like anxiety. They may fail to cope with the environment when they perceive that it is highly dynamic. In contrast, entrepreneurial team members with a low intolerance for uncertainty will not exaggerate the uncertainty that emerges from dynamic environments and be affected by negative emotions. They can positively cope with dynamic environments, engage in information exchange behavior to reduce feelings of uncertainty, and improve individual innovation. Hence, this study proposes the following:

**Hypothesis** **4.** *Among entrepreneurial team members, their intolerance for uncertainty can negatively moderate the indirect positive relationship between perceived environmental dynamism and individual innovation through information exchange behavior. This indirect positive relationship will weaken when entrepreneurial team members demonstrate a high intolerance for uncertainty.*

### 2.3. Team Cooperative Climate: A Moderating Effect

In addition to individual differences, organizational factors can also affect uncertainty-reducing behavior among individuals [9]. Team cooperative climate is a unique organizational factor that stresses interdependence and cooperation [23]. In teams with a high cooperative climate, members will be more willing to provide others with sincere and useful information. Therefore, individual’s information exchange behavior will have a better uncertainty reduction effect. Second, Previous studies posit that information sharing may reduce individuals’ relative resources, negatively affecting information exchange behavior [35,36]. In teams with high cooperative climate, members focus more on encouraging cooperation and mutual help [37], and do not overly stress about competitiveness. This reduces potential costs for team members to engage in information exchange behavior, and enables engagement when members perceive high environmental dynamism. Therefore, this study proposes the following:

**Hypothesis** **5.** *Entrepreneurial team cooperative climate can moderate the indirect positive relationship between members’ perceived environmental dynamism and individual innovation through information exchange behavior. This indirect positive relationship weakens when entrepreneurial teams demonstrate a low cooperative climate.*

## 3. Research Design

### 3.1. Sample Selection and Data Collection

We used questionnaire survey to test our hypothesis. Considering the particularity of our research goal, we applied purposive sampling. The questionnaire survey was conducted among entrepreneurial teams from three incubators in Beijing. All entrepreneurial teams are from the Internet industry. Thus, these teams can involve in dynamic environment and innovation. We used online questionnaire to collect data. The administrators of these incubators helped us to collected data. To prevent common method bias, data were collected across two periods with an interval of two weeks. In the first period, this study collected data on three variables, including perceived environmental dynamism, team cooperative climate, and individuals’ intolerance for uncertainty, as described by team members. Two weeks later, this study collected data on team members’ innovative behavior, as described by team leaders, and data regarding information exchange behavior, as depicted by team members.

One hundred and twenty team leader questionnaires and 484 team member questionnaires were collected. Three team leader questionnaires and five team member questionnaires were invalid (with incomplete data) and were excluded. Finally, 117 team leader questionnaires and 479 of their team members questionnaires were effective. Three to eight questionnaires were collected from team members in each entrepreneurial team. The average team size of these teams are 12.04 team members. Additionally, the average formation time of these teams are 11.24 months. The average age of team members is 28.62 and 47% are male.

### 3.2. Variable Measurement

This study used scales that has been widely adopted by overseas scholars and translates the original English scale into Chinese by strictly adhering to the translation method proposed by Brislin [38]. All variables are measured using the seven-point Likert scale, where one represents “Strongly Disagree” and seven represents “Strongly Agree.”

- Perceived external environmental dynamism: Drawing upon the scale proposed by Miller and Droge [39], this scale consists of five items, including “I think that our team has to constantly change marketing strategies to cope with market changes and external competition.” The Cronbach’s alpha is 0.91.
- Information exchange behavior: This variable is measured using the scale employed by Gong et al. [20], which consists of four items, including “I often exchange information with my team members and learn from them.” The Cronbach’s alpha is 0.93.
- Individual innovation: This study adopts the scale developed by Liu and Shi [40], which consists of six items, including “He/she often proposes innovative ideas at work.” The Cronbach’s alpha is 0.92.
- Intolerance for uncertainty: This variable is measured by using a simplified scale proposed by Carleton, Norton, and Asmundson [41]. It consists of 12 items, including “Unforeseen events make me feel very anxious.” The Cronbach’s alpha is 0.90.
- Team cooperative climate: This variable is measured using the scale proposed by Bogaert et al. [42], which consists of three items, including “In this team, cooperation is considered very important.” The Cronbach’s alpha is 0.93. This variable is a team-level variable that is aggregated from the scores of each entrepreneurial team member. Upon testing, the average Rwg of this variable is 0.85, while the median Rwg of this variable is 0.92. Since both are higher than the standard value of 0.7 that is adopted in general studies, this variable has a sufficient within-group consistency. The value of ICC (1) for this variable is 0.41. Based on Bliese’s [43] (recommendation, this variable meets the criteria of being greater than 0.05 and less than 0.5, indicating that it has large between-group differences. The value of ICC (2) for this variable is 0.73, which is greater than the standard value of 0.7, which further indicates that it has large between-group differences. ln summary, all three indicators above meet the requirements, indicating that team cooperative climate demonstrates a sufficient level of aggregation and agreement, where this variable can be aggregated.
- Control variables: At the level of individual variables, this study controls for entrepreneurial team members’ age, gender, and education levels. Regarding team variables, this study controls for entrepreneurial teams’ size and time of their formation, where the size of a team is measured by its number of stable employees.

## 4. Results

This study conducted the following steps to do the statistical analysis. First, we tested the reliability of variables (Cronbach’s alpha) and discriminant validity between variables (CFA). Second, we did descriptive statistical analysis of our sample including mean, standard deviation, and correlation. Third, because our data are nested with team and individual level variables, we used the cross-level path analysis to test hypotheses.

### 4.1. Confirmatory Factor Analysis

This study conducts a confirmatory factor analysis using Mplus 7.4, and the results of this analysis are shown in Table 1. This study fits a five-factor model (χ^2^ = 2106.27, df = 395, RMSEA = 0.03, TLI = 0.98, CFI = 0.98, SRMR = 0.02). According to the parameter criteria, this model fits the data well. As observed in Table 1, the chi-square value of the five-factor model is significant, and its parameter indicators perform better compared to the four-factor model and other models. This indicates that the fitting effect of other models is significantly worse than that of the five-factor model, while the five variables involved in this study have adequate discriminant validity.

### 4.2. Descriptive Statistical Analysis

Table 2 summarizes the mean, standard deviation, and correlation coefficient for each variable in this study. Perceived environmental dynamism among entrepreneurial team members has a positive correlation with their information exchange behavior (β = 0.57, *p* < 0.01), while the latter is positively correlated with individual innovation (β = 0.55, *p* < 0.01). Meanwhile, a positive correlation exists between the perceived environmental dynamism among entrepreneurial team members and their individual innovation (β = 0.30, *p* < 0.01). These findings preliminarily support some of the hypotheses posed in the above theoretical model.

### 4.3. Hypothesis Testing and Analysis

This study distinguishes the effects of different levels by using a cross-level path analysis method and performing hypothesis testing using the statistical software, Mplus. A model was constructed based on the above research hypotheses, and the parameters of model fitting were as follows: χ^2^ = 3.24 *** and df = 5; RMSEA = 0.01, which is lower than 0.05; CFI = 0.98 and TLI = 0.98, demonstrating a relatively adequate fit for the model. Based on the relatively satisfactory fit for the overall model, this study obtains the path coefficients for its direct and indirect effects, while also conducting path testing on moderating effects. These coefficients are shown in Table 3.

**Figure 2:**
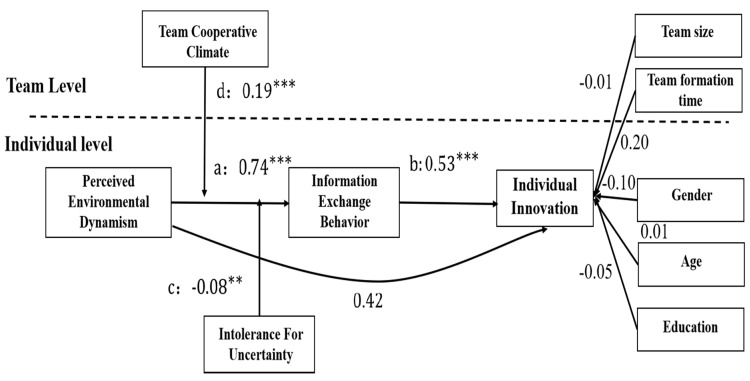
The model’s path diagram. Note: ** *p* < 0.01, *** *p* < 0.001.

Figure 2 illustrates this study’s path testing results. Hypothesis 1 suggests that perceived external environmental dynamism among entrepreneurial team members will positively affect information exchange behavior. According to Figure 2, the path coefficient for this effect is 0.74 (*p* < 0.005, 95% CI [0.349, 1.121]), which is significant. Therefore, Hypothesis 1 is supported.

Hypothesis 2 suggests that information exchange behavior will positively affect individual innovation. According to Table 3, the path coefficient for this effect is 0.53 (*p* < 0.005, 95% CI [0.308, 0.742]), which is significant. Therefore, Hypothesis 2 is supported.

Hypothesis 3 suggests that information exchange behavior can mediate the positive effect of perceived environmental dynamism on individual innovation. According to Table 3, the path coefficient for this indirect effect is 0.39 (*p* < 0.01, 95% CI [0.103, 0.669]), which is significant. Hypothesis 3 is supported.

**Figure 3:**
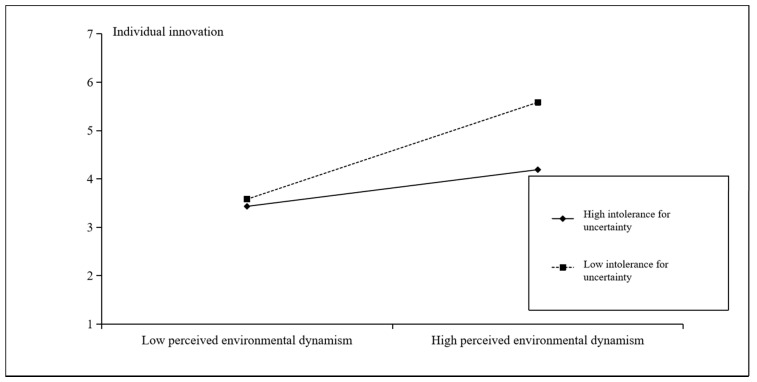
The moderating effect of intolerance for uncertainty.

Hypothesis 4 suggests that entrepreneurial team members’ intolerance for uncertainty will moderate the indirect positive relationship between their perceived environmental dynamism and individual innovation, which is mediated by information exchange behavior. Table 3 shows that the unstandardized path coefficient of the moderating effect with a mediator is −0.04 (*p* < 0.05, 95% CI [−0.076, −0.004]), which is significant. Therefore, Hypothesis 4 is supported. To better demonstrate the moderating effect of intolerance for uncertainty, this study illustrates the interaction—as shown in Figure 3—according to the recommendation of Cohen et al. [44]. Although the correlation coefficient’s change between the groups with a high and low intolerance for uncertainty among entrepreneurial team members is small (only −0.187), the correlation coefficients change significantly (*p* < 0.05). When entrepreneurial team members have a low intolerance for uncertainty, a stronger correlation exists between perceived environmental dynamism and individual innovation; when they have a high intolerance for uncertainty, perceived environmental dynamism has a lower positive effect on individual innovation.

**Figure 4:**
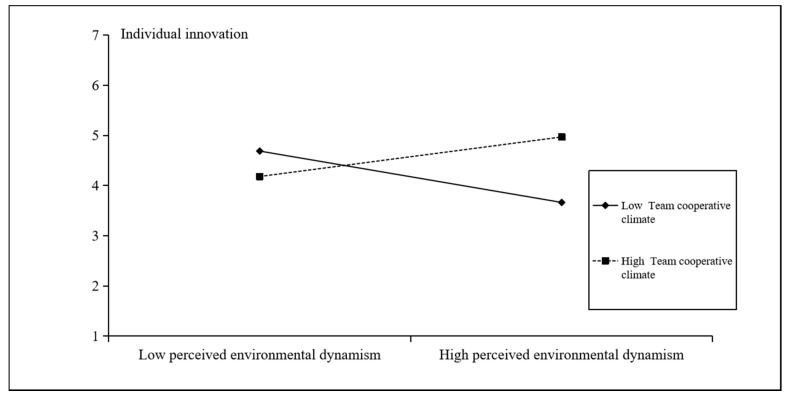
The moderating effect of team cooperative climate.

Hypothesis 5 suggests that team cooperative climate will moderate the indirect positive relationship between perceived environmental dynamism and individual innovation across levels, which is mediated by information exchange behavior. According to Table 3, the unstandardized path coefficient of the cross-level moderating effect with a mediator is 0.10 (*p* < 0.01, 95% CI [0.028, 0.185]). Therefore, Hypothesis 5 is supported. As shown in Figure 4, the correlation coefficients of perceived environmental dynamism and individual innovation between groups with varying levels of cooperative climate differ significantly, which is 2.86 (*p* < 0.005). When entrepreneurial teams have a strong cooperative climate, a stronger correlation exists between perceived environmental dynamism and individual innovation; when entrepreneurial teams have a weak team cooperative climate, perceived environmental dynamism has a lower positive effect on individual innovation.

## 5. Discussion

### 5.1. Theoretical Contributions

This study has made some theoretical contributions. First, although prior entrepreneurship studies have realized the influence of the uncertain environment on venture teams [45], few of them focuses on the effect of uncertain environment on individual level factors. This study has taken the lead in investigating the effect of perceived environmental dynamism on entrepreneurial team members’ innovation. It fills in the research gap of finding the influence of environmental dynamism on individual, broadening the existing entrepreneurship research.

Second, to explore how dynamic environment can influence organizational behaviors, the existing environment research usually starts with a strategic perspective [46,47]. However, the strategic perspective cannot be applied to the individual level. This study uses the uncertainty reduction theory, which introduces a new perspective. It not only explains the relationship, but also broaden the scope of application of the uncertainty reduction theory.

Lastly, this study reveals the whole mechanism that impose the effect of uncertain environment on member’s innovation, where information exchange behavior is the mediator and the intolerance for uncertainty and team cooperative climate are the moderators. It further enriches research on the general effects of environmental dynamism in entrepreneurial teams on individual innovation.

### 5.2. Practical Implications

This study’s results have practical implications for entrepreneurial team to improve innovation under dynamic environment. First, the results suggest team member’s information exchange behavior is the antecedents of member’s innovation, which indicates that boosting this behavior of members is more likely to improve innovation. Practically, organizations in dynamic environment can encourage and give support to team member’s information exchange behavior [48,49]. Second, our results support the hypothesis that high team cooperative climate and high individual intolerance for uncertainty can strengthen the positive relationship between perceived environmental dynamism and individual innovation. Thus, we suggest that team leaders can build up cooperative climate in the team and select team members with the personality of the intolerance of uncertainty, when the external environment is dynamic.

### 5.3. Limitations

This study has some limitations. First, although the questionnaires were collected at different times, they were completed by entrepreneurial team members, so the common method bias is unavoidable. Future research may collect data from multiple sources or employ other research methods to reduce the common method bias. Second, all of the samples come from Beijing and Internet-related industries. Although the possible effects of regional and industry factors have been reduced, this sample selection may influence the representativeness of the conclusions. In the future, the scope of research can be further expanded to reduce bias. Third, this study only focuses on the positive effects of environmental dynamism on individual innovation. In fact, a dynamic environment also entails a higher risk [21], so it is worthwhile to investigate the potential negative effects on individual innovation and other team behaviors. Lastly, according to feedback from previous field interviews, this study uses the information exchange behavior as the primary means for reducing uncertainty among entrepreneurial team members. Since individuals also adopt other uncertainty-reducing methods, however, future research can enrich and expand the topic from this perspective [50].

## 6. Conclusions

Based on the uncertainty reduction theory, this study utilizes empirical research to investigate the effect and boundary conditions of perceived environmental dynamism among entrepreneurial team members on their individual innovation behavior [51]. The results showed that perceived environmental dynamism positively affected their individual innovation through their information exchange behavior. When entrepreneurial team members have a high intolerance for uncertainty, the indirect positive relationship between perceived environmental dynamism and individual innovation (via information exchange) is weakened [52]. When their team have a high cooperative climate, the indirect positive relationship between perceived environmental dynamism and individual innovation (via information exchange) among entrepreneurial team members is strengthened.

## Figures and Tables

**Table 1:** Results of the confirmatory factor analysis.

Description	χ^2^	df	RMSEA (90% CI)	TLI	CFI	SRMR
Five-factor model (hypothetical model)	582.27 ***	395	0.03 (0.026, 0.037)	0.98	0.98	0.02
Four-factor model (perceived environmental dynamism and intolerance for uncertainty combined)	2126.35 ***	399	0.10 (0.091, 0.099)	0.86	0.87	0.12
Three-factor model (perceived environmental dynamism, intolerance for uncertainty, and team cooperative climate combined)	3272.74 ***	402	0.12 (0.118, 0.126)	0.77	0.80	0.13
Two-factor model (perceived environmental dynamism, intolerance for uncertainty, team cooperative climate, and information exchange behavior combined)	4728.04 ***	404	0.15 (0.146, 0.153)	0.66	0.68	0.17
One-factor model (all combined)	8120.14 ***	405	0.20 (0.196, 0.203)	0.40	0.44	0.22

*** *p* < 0.001.

**Table 2:** Variables and coefficients: Descriptive statistics of a correlation between variables.

Variable	Mean	Standard Deviation	1	2	3	4	5	6	8	9
**Individual level**										
1. Gender	0.67	0.47								
2. Age	28.62	5.89	−0.09							
3. Education	2.24	0.72	0.04	0.32 **						
4. Perceived environmental dynamism	4.96	0.95	0.05	−0.07	0.02					
5. Information exchange behavior	5.47	1.21	0.03	−0.04	0.01	0.57 **				
6. Individual innovation	5.12	1.40	0.00	−0.04	−0.04	0.30 **	0.55 **			
7. Intolerance for uncertainty	4.71	1.49	−0.03	−0.05	−0.02	−0.03	−0.22 **	−0.22 **		
**Team level**										
8. Team size	12.04	5.21								
9. Team formation time	11.24	3.18							0.14 **	
10. Team cooperative climate	4.11	1.17							0.21 **	0.21 **

Note: The number of individual-level samples = 479; the number of team-level samples =117, ** *p* < 0.01.

**Table 3:** The results of unstandardized path coefficients.

Structural Path	Unstandardized Path Coefficient
H1: Perceived environmental dynamism →information exchange behavior	a: 0.74 (95% CI: 0.349, 1.121)
H2: Information exchange behavior → individual innovation	b: 0.53 (95% CI: 0.308, 0.742)
H3: Perceived environmental dynamism →information exchange behavior → individual innovation	A × b: 0.39 (95% CI: 0.103, 0.669)
Moderating effect of intolerance for uncertainty on H1	c: −0.08 (95% CI: −0.133, −0.019)
Individual level: Information exchange behavior →individual innovation	b: 0.53 (95% CI: 0.308, 0.742)
H4: Moderating effect of intolerance for uncertainty on (perceived environmental dynamism →information exchange behavior → individual innovation) with a mediator	c × b: −0.04 (95% CI: −0.076, −0.004)
Moderating effect of team cooperative climate on perceived environmental dynamism at team level →information exchange behavior	d: 0.19 (95% CI: 0.103, 0.279)
Individual level: Information exchange behavior →Individual innovation	b: 0.53 (95% CI: 0.308, 0.742)
H5: Moderating effect of team cooperative climate on (perceived environmental dynamism → information exchange behavior → individual innovation) with a mediator	d × b: 0.10 (95% CI: 0.028, 0.185)
